# The Validation of Surgical Simulators: A Technical Report on Current Validation Terminology as a Reference for Future Research

**DOI:** 10.7759/cureus.31881

**Published:** 2022-11-25

**Authors:** Thomas Hughes, Joseph T Fennelly, Rakesh Patel, Jonathan Baxter

**Affiliations:** 1 Trauma and Orthopaedics, Arrowe Park Hospital, Wirral, GBR; 2 Trauma and Orthopaedics, Leighton Hospital, Crewe, GBR; 3 Nephrology, Queens Medical Centre, Nottingham, GBR; 4 Trauma and Orthopaedics, Stoke Mandeville Hospital, Aylesbury, GBR

**Keywords:** simulation education, validation, simulation in medical education, surgical training, terminology

## Abstract

In recent years, surgical trainees have been exposed to a lower volume of operative procedures. In part, this is due to the reduction in working hours and further disruption by the coronavirus disease 2019 pandemic. Much has been done to develop the techniques of surgical skill training outside of the operating theatre. Simulation-based interventions must undergo a process of validation to assess their appropriateness and effectiveness for use in training. The terminology of validation within current literature, however, has not evolved in line with the education community, resulting in varying definitions for the same phrase across domains. This can result in confusion and misinterpretation among researchers and surgeons working within this domain. This technical report describes the “types of validity” definitions used in the traditional framework of surgical simulation literature and the contemporary, unitary framework of validity adopted by educationalist theorists. There is a clear overlap between the traditional “types of validity” and the contemporary, unitary framework. The divergence in the use of those definitions seems, at least partly, influenced by the context of the investigations being conducted. By utilising the contemporary definitions, authors may have struggled to provide the evidence required to justify the use of the multitude of surgical skill simulators developed in the recent past. This report has provided an overview of the current terminology within the validation frameworks and can be used as a reference for future surgical simulation research.

## Introduction

Surgeons in training are being exposed to a lower volume of surgical procedures with fewer opportunities to develop the psychomotor skills required for complex tasks such as arthroscopic surgery [1]. In part, this is due to a reduction in hours worked by junior doctors following the introduction of the European Working Time Directive in combination with the overall shortening in the length of training seen with the Calman reforms [2]. In response to the recent coronavirus disease 2019 pandemic, training opportunities were temporarily reduced due to cancellations in elective operating and trainees being mobilised to work in other specialities [3-5]. Consequently, much has been done to develop techniques for surgical skill training outside of the operating theatre [6]. One growing area of research is in the use of simulation-based intervention (SBI) in surgery [7]. Simulation is a versatile training modality that has the potential for use in the development of surgical skills, assessment, candidate selection, and practice of complex procedures or challenging steps within such procedures [8].

The overarching goal in surgical skill simulation is to provide a learning environment in which the psychomotor skills required for surgical practice can be learnt and developed safely and efficiently and ultimately utilised in the operating theatre. Before a surgical skill SBI is utilised for training purposes, it should undergo an assessment of appropriateness for its use in this way. This is often referred to as a process of validation [9]. The concept and terminology of validation with reference to surgical SBI research, however, have not evolved in line with the education community, resulting in varying definitions for the same phrases across domains [10]. This paper summarises the “types of validity” used in the traditional framework of surgical simulation literature and the contemporary, unitary framework of validity adopted by educationalist theorists. By doing so, this report aims to (1) provide a clear overview of current validation terminology as a reference for future research, and (2) discuss the future directions for the validation of SBI research.

## Technical report

The traditional validity framework

The traditional framework uses the concept of “types of validity” (content, face, construct, transfer/concurrent) and these definitions continue to be used extensively in the surgical simulation literature (Table 1) [11]. The definition of construct validity, in particular, has evolved notably since its inception [12]. The original concept was to link contexts with intangible attributes and no definitive criterion (constructs) to observable attributes in order to be measured. An example of such an entity is surgical expertise. Validity could then be investigated by assessing these attributes and their theorised relationships to the original context in question [13].

**Table 1:** Traditional “types of validity” as commonly used in surgical simulation research. [11].

Type of validity	Description
Construct	The simulator can accurately differentiate candidates based on relative surgical skill; this represents the ability of a simulator to be used as an assessment tool
Face	The impression of how realistic a simulation feels (“haptic”) and looks; this concept includes the acceptability of a simulator to the trainee
Content	The simulation contains the important components of a task as assessed by an expert; this represents the educational content of a simulator
Transfer/Concurrent	Training on a simulator improves performance in the operating theatre; this represents the effect of simulation on real-world performance

The concept of face validity as an independent entity has somewhat fallen out of favour in the educational community [14,15]. It is still used in the surgical simulation literature but with varying definitions, methods of assessment, and conclusions. Any SBI being designed for use in training or assessment must strike a balance between what is educationally and scientifically superior and what is acceptable to the trainee. The general description of face validity incorporates this concept by assessing how realistic the simulator looks and feels [11]. This provides a source of evidence as to the acceptability of the simulator to the intended user although is conditional to an appropriate sample of participants being selected during the assessment process.

Content validity, as defined in Table 1, describes the association between the components of a test and the task that it purports to represent. This is ascertained largely through expert opinion. Qualitative data regarding face validity in surgical simulation research are largely obtained through the use of subjective questionnaires [16]. These concepts can be thought of to represent the acceptability of the simulator to both the trainee and the expert and represent an important source of evidence when developing a validation argument for the use of SBI.

The ultimate goal of SBI is to improve surgical performance during real-life surgery. Observing improved performance during real-life surgery following training on an SBI provides evidence for transfer validity [17].

The contemporary validity framework

More recently, it has been noted that the different “types of validity,” in combination with measured metrics, ultimately resulted in a common pathway supporting or refuting the relationship to the construct [10]. As a result, educational theorists abandoned the concept of “types of validity” and adopted a contemporary, unitary framework. This evolution in terminology has been universally accepted within the educational community; however, this has not been reflected in the methodology of surgical skill simulation research [18,19].

In the contemporary framework, construct validity is the only form of validity and the study of such requires justification of the sources of evidence to support the interpretation of whether the instrument achieves what it purports to [9]. The concept of validity using this contemporary framework is a property of inferences, not just the instrument, and, therefore, a validation argument must be established for each intended interpretation [20]. The five sources of evidence specified in the contemporary, unitary framework include (1) content, (2) response process, (3) internal structure, (4) relation to other variables, and (5) consequences [21]. The sources of evidence and their definitions are summarised in Table 2.

**Table 2:** Contemporary definitions of validity used in surgical simulation validation studies. [19].

Validity evidence source	Definition
Content	The relationship between a test’s content and the construct it is intended to measure
Response process	Analysis of responses (actions, strategies, thought processes) of individual respondents or observers. Differences in response processes may reveal sources of variance irrelevant to the construct being measured. It includes instrument security, scoring, and reporting of results
Internal structure	The degree to which individual items within an instrument fit the underlying constructs. It is often reported by measures of internal consistency, reliability, and factor analysis
Relations to other variables	Relationship between scores and other variables relevant to the construct being measured. Relationships may be positive (convergent/predictive) or negative (divergent/discriminant) depending on the constructs being measured
Consequences	Assessments are intended to have some desired effect or may have unintended effects

Content evidence pertains to the relationship between the test content and the construct of interest. Evidence to this effect is acquired by seeking the opinion of those with expertise in the appropriate content domain, who may then design a blueprint that is representative of the desired construct such as surgical skill. This represents an expert opinion as to the relationship between the content of the test and the content domain of the construct.

The response process refers to the integrity of the data collected as evidence of the test administration. It mandates that sources of error are controlled for or eliminated to the maximum extent possible through the appropriate handling of acquired data, be it responses or assessment scores.

Internal structure refers to the source of the validity evidence relating to the appropriate statistical or psychometric characteristics of the assessment tool. This is often termed reliability and encompasses the reproducibility and generalisability of the results.

Relationship to other variables encompasses the correlation between the measured score and score of appropriate criterion measures [9]. This source of evidence includes the traditional concept of construct validity that describes the correlation between performance scores on the simulated activity in question and the level of expertise, be it the number of procedures performed or the level of training. Although this is an important source of evidence, its use in isolation may lead to false conclusions being drawn regarding the overall usefulness of a given simulator performance metric in assessing a construct.

An understanding of the consequences to the learner affords further evidence to the overall validity of the inferences made in assessing the construct with the use of the SBI. This aspect is somewhat controversial relative to the other concepts, but the importance as a source of evidence and the need to investigate and report upon it is generally agreed upon [19].

## Discussion

One obstacle encountered during contemporary research on SBI is rationalising the discrepancy between the terminology used to describe the different facets of validation in the educational and surgical simulation literature. A review conducted by Ghaderi et al. in 2015 highlighted a considerable discrepancy between the contemporary framework for conceptualising validity preferred by medical educators and that adopted by both surgical educators and authors of studies into the use of simulation in surgical skill training [19]. This review builds on the concerns of Korndorffer et al. who in 2010 identified that the accepted framework for validity had not been embraced by the surgical community [18]. They identified 47 studies validating laparoscopic simulation, all utilising the “outdated” framework of “types of validity,” 75% construct, 38% face, and 11% content.

There is a clear overlap between the traditional “types of validity” and the contemporary, unitary framework. It is interesting to contemplate why there has been such a universal lack of uptake of the contemporary framework within the surgical education fraternity. With an understanding of the contemporary framework and the terminology thereof, one can see how the use of multiple sources of evidence can shape the overall understanding of the inferences made regarding measured performance on an SBI and provide evidence supporting the validity argument of these interpretations.

Part of the appeal of the traditional definitions of “types of validity,” as outlined in Table 1, are that they lend themselves more readily to scientific investigation. Through the utilisation of such discrete terminology, authors have been able to design investigations to prove or disprove the existence of each different “type of validity,” a concept that is considered inaccurate according to the contemporary approach [20].

The divergence in the use of those definitions drawn up by educationalists and psychology experts and those utilised by surgical educators seems, at least partly, influenced by the context of the investigations being conducted. By utilising the contemporary definitions, authors may have struggled to provide the evidence required to justify the use of the multitude of surgical skill simulators developed in the recent past [18,20]. This would make it difficult to justify their inclusion in surgical skill training programmes, which is one of the overarching goals in the development of such simulators. However, this does not imply that the explicit use of the contemporary framework is entirely more appropriate for this task. If the design and implementation of investigations into the usefulness of surgical simulators are hindered by the complexity of the sources of validity evidence required by the contemporary framework, then the evidence behind the appropriateness of their use may well never be acquired at all. With the extent of the use of the traditional “types of validity” in the surgical simulation literature to date, it is likely that further studies will adopt these definitions to maintain consistency. It is, therefore, possible that the apposite way forward is a compromise involving future authors clearly stating the definitions used and their justification thereof even if the choice is to utilise the traditional “types of validity.”

## Conclusions

When interpreting the current literature, one should have a broad understanding of the two main frameworks and that any inferences made are placed in the appropriate context. This technical report has provided an overview of the current terminology within the validation frameworks. We hope this can be used as a reference for future research into SBI validation and ultimately support the progression of surgical skill training outside of the theatre.
